# *XRCC1* Arg399Gln and Arg194Trp Polymorphisms and Risk of Systemic Lupus Erythematosus in an Iranian Population: A Pilot Study

**DOI:** 10.1155/2014/492956

**Published:** 2014-05-26

**Authors:** Saeedeh Salimi, Milad Mohammadoo-khorasani, Ehsan Tabatabai, Mahnaz Sandoughi, Zahra Zakeri, Anoosh Naghavi

**Affiliations:** ^1^Cellular and Molecular Research Center, Zahedan University of Medical Sciences, Zahedan, Iran; ^2^Department of Clinical Biochemistry, School of Medicine, Zahedan University of Medical Sciences, Zahedan, Iran; ^3^Department of Internal Medicine, School of Medicine, Zahedan University of Medical Sciences, Zahedan, Iran

## Abstract

*Background*. Evidences are suggesting that DNA damage is implicated in development of systemic lupus erythematosus (SLE). Therefore we focused on two common *XRCC1* polymorphisms (Arg399Gln and Arg194Trp) in SLE susceptibility in South East of Iran. *Methods*. Peripheral blood DNA was extracted from 163 SLE patients and 180 healthy controls. PCR-restriction fragment length polymorphism method was used for genotyping of *XRCC1* Arg399Gln and Arg194Trp polymorphisms. *Results*. The frequency of Arg/Gln genotype of the *XRCC1* Arg399Gln polymorphism was significantly lower in SLE patients than controls. Moreover, lower frequency of Arg/Gln genotype was found in SLE patients with malar rash compared to patients without this manifestation. No association was observed between *XRCC1* Arg194Trp polymorphism and increased risk of SLE in studied population. Haplotype analysis revealed no correlation between four haplotypes of *XRCC1* Arg399Gln and Arg194Trp polymorphisms and SLE risk. *Conclusion*. These findings suggest that *XRCC1* 399 Arg/Gln heterozygous genotype plays a protective role in SLE susceptibility.

## 1. Introduction

Systemic lupus erythematosus (SLE) is a multisystemic and autoimmune disease that is characterized principally by the increasing of B-cells activity and autoantibodies production against self-antigens. [1–3]. SLE is tenfold more common in women in reproductive age and its prevalence varies with race/ethnicity, geography, and socioeconomic status [4, 5]. Although the pathogenesis of SLE remains elusive, the etiology of this disease is supposed to involve genetic, hormonal, and environmental factors [6]. Diverse autoantigens are considered as targets of autoantibodies in SLE [1]. Antigenicity may be increased by reactive oxygen species (ROS), chemicals, drugs, and ultraviolet (UV) exposure and, therefore, changes DNA conformation, which results in DNA base damage and breaks [7, 8].

There are a variety of autoantibodies to intracellular proteins and nucleic acids in SLE patients' sera such as dsDNA autoantibody which may correlate with disease activity and manifestations [7]. Some studies have shown that impairment of DNA repair system leads to accumulation of breaks in DNA. DNA breaks combined with nuclear proteins may cause immunogenic antigens, and this induces the production of autoreactive antibodies (Abs) and autoimmune response in susceptible individuals [9].

DNA repair enzymes ceaselessly checked the chromosomes to correct damaged nucleotide produced by methylation, oxidation, or oxidative damage [10]. Base excision repair (BER) and nucleotide excision repair (NER) are two pathways that repair most of the DNA damages including ROS [11]. The X-ray repair cross-complementation group 1 (XRCC1) is one of the most important proteins that has important role in the BER pathway [12]. This DNA repair protein is responsible for the effective repair of DNA damage caused by active oxygen, ionization, and alkylating agents [13]. The 33 kb human* XRCC1* gene is located on chromosome 19q13.2-13.3 and has 17 exons [14]. There are more than 300 valid single nucleotide polymorphisms in the* XRCC1* gene mentioned in the dbSNP database (http://www.ncbi.nlm.nih.gov/SNP), in which three of them are more frequent [15] and lead to amino acid replacement: Arg194Trp (exon 6, C to T substitution, and rs1799782), Arg280His (exon 9, G to A substitution, and rs25489), and Arg399Gln (exon 10, G to A substitution, and rs25487). Although the functional effects of these polymorphisms in* XRCC1* have not been understood, it is suggested that amino acid changes at preserved regions may alter its function [16]. This change in protein biochemistry leads to the hypothesis that variant alleles may reduce kinetics repair, thereby resulting in SLE susceptibility [17].

Although several evidences have shown the association between polymorphisms of* XRCC1* gene and autoimmune diseases, there are few published reports about the association between* XRCC1* Arg399Gln and Arg149Trp polymorphisms and SLE.

Since XRCC1 plays important role in DNA repair, we studied the functional polymorphisms of* XRCC1* gene in association with SLE risk in an Iranian population.

## 2. Materials and Methods

### 2.1. Patients and Sample Collection

In this case-control study, all SLE patients (163 patients, 13 men and 150 women) were recruited from Department of Rheumatology of Ali ibn Abi Talib Hospital (Zahedan, Iran), since 2011 to 2013. Individuals with other rheumatic diseases, infections, or malignant tumors were excluded from the study. All patients fulfilled at least four items of systemic lupus erythematosus according to ACR 1997 criteria (American Rheumatology Association). A written informed consent was obtained from all participants.

The control group consisted of 180 unrelated age, sex, and ethnicity matched individuals (14 men and 166 women) with negative ANA test. They were randomly selected from subjects who were referred to internal medicine clinic for checkup examinations. The controls had no systemic disease and family relation with lupus patients.

All participants provided written informed consent according to the Declaration of Helsinki. The project was approved by the Zahedan University of Medical Sciences Ethics Committee.

### 2.2. Genomic DNA Extraction and Genotyping

Blood samples were collected in 2 mL Na-EDTA tubes from patients and healthy controls and genomic DNA was extracted by using salting out method.

Tetraprimer amplification refractory mutation system (ARMS) was designed for detection of* XRCC1* Arg399Gln and Arg194Trp polymorphisms.

This method is rapid, simple, and economical for SNP analysis based on allele-specific primers. In this method four primers are necessary to amplify a larger fragment from template DNA comprising the SNP and two smaller fragments representing each of the two allele-specific products. The genomic sequences of genes were obtained from the National Center for Biotechnology Information (NCBI) (http://www.ncbi.nlm.nih.gov). For each polymorphism, we used two external primers (Forward outer and Reverse outer) as an internal control to check the performance of PCR and two allele-specific internal primers that were designed in opposite orientation (Forward inner and Reverse inner) for detection of each allele. The allele-specific amplicons have different lengths and can be separated easily by standard gel electrophoresis.

The tetraprimer ARMS-PCR primer sequences, the annealing temperature, and the amplicon sizes are listed in Table 1.

The cycling conditions were 6 min at 95°C followed by 35 cycles of 95°C for 30 s, annealing temperature as in Table 1 for 30 s, 72°C for 30 s and a final cycle 72°C for 6 min. PCR products were separated by standard electrophoresis on 2.5% agarose gel containing ethidium bromide.

All samples were analyzed by PCR-restriction fragment length polymorphism (PCR-RFLP) using* Msp I *restriction enzyme (Fermentas, Lithuania), as previously described by Ryu et al. for quality control [18].

### 2.3. Statistical Analysis

Statistical analysis was performed with SPSS-V-18. Student's *t*-test was used to compare quantitative variables and *χ* ^2^ test was used to compare nonquantitative variables. The independent effect of each risk polymorphism on SLE was examined by logistic regression analysis. Linkage disequilibrium (LD) was analyzed using CubeX software [19]. Values of *P* < 0.05 were considered statistically significant.

## 3. Results

Demographic data of SLE patients and control group are shown in Table 2. There were no significant differences in gender and ethnicity between SLE patients and controls. Moreover, there was no considerable difference in the mean age between control group (32.1 ± 11.7) and SLE patients (32.6 ± 8.6; *P* = 0.4).

According to our findings dermomucus manifestations developed in 85% (83) of SLE patients (54% with malar rash). Arthritis was found in 84% (87) of patients, whereas neuropsychiatric manifestations were observed in 17% of patients. Lupus nephritis was developed with raised serum creatinine in 27% of patients. According to the main laboratory features, 58% presented anti-phospholipid antibody syndrome (APS) and 14% presented persistent leucopenia.

The allele and genotype frequencies of SLE patients and controls are shown in Table 3. All loci were in the Hardy-Weinberg equilibrium.

Linkage disequilibrium was calculated for* XRCC1* gene polymorphisms (rs1799782 and rs25484) for the whole population (*D*′ = 0.708; *r* ^2^ = 0.162).

The genotypes and alleles frequency of* XRCC1* Arg194Trp (rs1799782) polymorphism were not statistically different between SLE patients and control group. However, the genotypic and allelic frequencies of* XRCC1* Arg399Gln (rs25484) polymorphism were statistically different between the two groups. The risk of SLE was lower in individuals with Arg/Gln and Gln/Gln genotypes in comparison to those with Arg/Arg genotype (OR = 0.44 [95% CI = 0.27 to 0.71]; *P* = 0.001 and 0.73 [95% CI = 0.5 to 1.02]; *P* = 0.06). In addition, the frequency of Gln allele was significantly lower in SLE patients (OR = 0.7 [95% CI = 0.5 to 0.94]; *P* = 0.02).

When the association between* XRCC1* polymorphisms and SLE manifestations was analyzed, we found higher frequency of* XRCC1* Arg399Gln Arg/Gln genotype in SLE patients with malar rash compared to SLE patients without this manifestation (OR = 0.42 (95% CI = 0.21–0.83); *P* = 0.01) (Table 4).

Moreover, no association was found between* XRCC1* polymorphisms and other SLE manifestations. NO differences in genotypes and alleles frequency of* XRCC1* gene polymorphisms were observed between the Balouch and Persian ethnic groups as well (*P* > 0.05).

Four haplotypes of the* XRCC1* gene consisting of two alleles of each polymorphism are shown in Table 5. There were not any differences in haplotype frequency between SLE patients and controls too.

## 4. Discussion

In the present study, our findings show that Arg/Gln genotype of* XRCC1* Arg399Gln polymorphism is a protective factor in SLE susceptibility; however there is no association between* XRCC1* Arg194Trp polymorphism and SLE. Moreover, lower frequency of Arg/Gln genotype of* XRCC1* Arg399Gln is observed in patients with malar rash compared to patients without this manifestation.

Evidences indicated that DNA damage and nucleoprotein accumulation may produce immunogenic antigens that induce the autoreactive antibodies production. DNA damage can be originated from environmental factors like mutagenic elements and UV exposure or internal factors including reactive oxygen species resulting from physiological process [20]. So far, extensive studies were performed on the role of oxidative stress in the immune system which causes various diseases and DNA structure alteration [21].

In 1991 Benke and Belmar indicated that DNA repair is diminished in patients with SLE [22]. Some other studies have shown that DNA repair was impaired in SLE patients; therefore, different polymorphic variants of genes encoding the proteins responsible for DNA repair could be involved in disease susceptibility and manifestations [23, 24].

DNA repair efficiency in cells is a determining factor for preventing the development of SLE [25, 26]. Although there are several reports about the correlation between* XRCC1* gene polymorphisms and autoimmune diseases such as rheumatoid arthritis and SLE [27, 28], data on allelic variation of* XRCC1* gene polymorphisms in SLE patients are limited. In 2009, Lin et al. analyzed the relationship between* XRCC1* Arg194Trp and Arg399Gln polymorphisms and SLE in the Taiwanese Han Chinese population and observed higher frequency of Arg/Gln genotype of Arg399Gln gene polymorphism in SLE patients [21]. In another study Bassi et al. found no correlation between* XRCC1* Arg399Gln,* XRCC3* Thr241Met, and* XRCC4* Ile401Thr polymorphisms and SLE; however, the* XRCC1*Gln/Gln or Arg/Gln genotypes were associated with the presence of anti-dsDNA antibody in Brazilians [29].

Recently, Warchoł et al. reported that carriage of Gln allele (Gln/Gln or Gln/Arg genotypes) of* XRCC1* Arg399Gln polymorphism is associated with SLE in Polish Population [30]. The correlation between Gln allele and malar rash or photosensitivity manifestations of SLE has been observed too. When they performed a meta-analysis on Brazilian, Taiwanese Han Chinese, and Polish populations, they concluded that the Gln/Gln or Gln/Arg genotypes and Gln allele were associated with SLE susceptibility. These results are completely inconsistent with our findings, because we observed lower frequency of the Arg/Gln genotype of* XRCC1* Arg399Gln polymorphism in SLE patients compared to control group and Arg allele is a risk factor in SLE susceptibility.

In consistent with our observations Yosunkaya et al. showed that the frequency of G allele of* XRCC1* Arg399Gln polymorphism was significantly higher in rheumatoid arthritis (RA) patients and carriers of Arg allele have greater risk of RA in Turkish patients [28].

Our study failed to show any significant differences in allelic and genotypic distribution of* XRCC1* Arg149Trp polymorphism among two groups. In accordance with our results, Lin et al. [21] found no association between the* XRCC1* Arg194Trp and susceptibility to SLE. Also, Koyama et al. did not find any association between* XRCC1* Arg194Gln and Arg399Gln polymorphisms and rheumatoid arthritis in Japanese population [27].

In conclusion, we found lower frequency of* XRCC1* Arg399Gln Arg/Gln genotype in SLE patients compared to healthy controls. There was no association between* XRCC1* Arg194Trp polymorphism and SLE susceptibility in South East Iranian population.

## Figures and Tables

**Table 1:** Designed primers for tetraprimer ARMS-PCR reactions, annealing temperature, and fragments sizes.

SNP	Primer sequence	Annealing temperature (°C)	Fragments length
*XRCC1 * Arg194Trp rs1799782	FO: 5′-CGTCCCAGGTAAGCTGTAC-3′ RO: 5′-CACTCCTATCTATGGGACACAG-3′ FI: 5′-CGGGGGCTCTCTTCTTCATCC-3′ RI: 5′-CACCTGGGGATGTCTTGTTGATACA-3′	63	Outer: 471 Arg: 297 Trp: 219

*XRCC1* Arg399Gln rs25487	FO: 5′-ACCAGCTGTGCCTTTGCCAACACC-3′ RO: 5′-CTGGAGTACCCCAGCCCCTGCC-3′ FI: 5′-GTCGGCGGCTGCCCTCACA-3′ RI: 5′-TGGCGTGTGAGGCCTTACCACC-3′	68	Outer: 283 Arg: 183 Gln: 140

**Table 2:** Demographic characteristics of SLE patients and controls.

Parameter	SLE patients, *n* = 163	Controls *n* = 180	*P* value
Age (years)	32.6 ± 8.6	32.1 ± 11.7	0.68
Sex, *n* (male/female)	13/150	14/166	0.6
Ethnicity, *n* (%)			0.36
Persian	82 (50)	86 (48)	
Balouch	81 (50)	94 (52)	

**Table 3:** Genotypes and alleles frequency of *XRCC1* polymorphisms in SLE patients and controls.

Polymorphism	SLE patients, *n* = 163	Controls, *n* = 180	*P* value	OR (95% CI)
Arg399Gln				
Genotypes, *n* (%)				
Arg/Arg	64 (39.3)	41 (22.8)		1
Arg/Gln	75 (46)	110 (61.2)	0.001	0.44 (0.27–0.71)
Gln/Gln	24 (14.7)	29 (16)	0.063	0.73 (0.5–1.02)
Alleles, *n* (%)				
Arg	203 (62.2)	192 (53.3)		1
Gln	123 (37.7)	168 (46.7)	0.02	0.7 (0.5–0.94)
Arg194Trp				
Genotypes, *n* (%)				
Arg/Arg	106 (65)	112 (62.2)		1
Arg/Trp	52 (32)	65 (36.1)	0.47	0.85 (0.54–1.3)
Trp/Trp	5 (3)	3 (1.7)	0.45	1.3 (0.64–2.75)
Alleles, *n* (%)				
Arg	264 (81)	289 (80.3)		1
Trp	62 (19)	71 (19.7)	0.8	0.96 (0.65–1.4)

**Table 4:** The association between *XRCC1* Arg399Gln polymorphism and malar rash in SLE patients.

Genotypes	SLE patients with malar rash, *n* (%)	SLE patients without malar rash, *n* (%)	*P* value	OR (95% CI)
Arg/Arg	41 (47)	23 (30)		Ref = 1
Arg/Gln	32 (37)	43 (57)	0.01	0.42 (0.21–0.83)
Gln/Gln	14 (16)	10 (13)	0.6	0.89 (0.55–1.4)

Total	87	76		

**Table 5:** Haplotypes frequency of *XRCC1* Arg399Gln and Arg194Trp polymorphisms in SLE patients and control group.

Haplotype Arg0399Gln-Arg194Trp	SLE patients	Controls	*P* value	OR (95% CI)
Arg-Arg(GC)	0.231	0.263		1
Arg-Trp(GT)	0.145	0.16	0.95	1 (0.5–2)
Gln-Arg(AC)	0.578	0.544	0.5	1.1 (0.8–1.4)
Gln-Trp(AT)	0.044	0.031	0.4	1.2 (0.8–1.7)
